# FOOD INTAKE WAS TEMPORALLY ASSOCIATED WITH STAFF MEALTIME CARE APPROACHES AND BEHAVIORS OF RESIDENTS WITH DEMENTIA

**DOI:** 10.1093/geroni/igad104.1895

**Published:** 2023-12-21

**Authors:** Wen Liu, Yelena Perkhounkova, Maria Hein, Roger Bakeman

**Affiliations:** The University Of Iowa, Iowa City, Iowa, United States; University of Iowa, Iowa City, Iowa, United States; University of Iowa, College of Nursing, Iowa City, Iowa, United States; Georgia State University, Atlanta, Georgia, United States

## Abstract

Nursing home residents with dementia commonly experience low food intake, resulting in functional and nutritional consequences. Prior work supports associative relationships of resident behaviors and staff approaches with food intake, yet research on temporal relationships is limited. This study examined temporal associations of resident behaviors and staff approaches with food intake. Videotaped mealtime observations (N=160) involving 36 staff and 27 residents (53 staff-resident dyads) in 9 nursing homes were analyzed. Sequential analysis was conducted for resident behaviors and staff approaches as antecedents of resident successful food intakes in 5-, 10-, and 15-second time windows. Increased resident-initiated food intake was associated with preceding resident positive verbal behaviors, but not other resident behaviors or staff approaches. Decreased resident-initiated food intake was associated with preceding resident functional impairments and resistive behaviors, but not other behaviors or approaches. Increased staff-initiated food intake was associated with preceding staff person-centered verbal approaches, but not resident behaviors or other staff approaches. Decreased staff-initiated food intake was associated with preceding resident positive/neutral nonverbal behaviors and functional impairments, and staff task-centered care approaches. Findings confirm the importance of certain strategies in promoting food intake, including facilitating resident positive verbal communication, supporting functional impairments, responding to resistive behaviors, maximizing the use of person-centered care, and minimizing the use of task-centered care. Resident positive/neutral nonverbal behaviors which reflect resident active engagement during mealtime, do not facilitate staff-initiated food intake and may be an indicator of resident independence and readiness to initiate food intake. Further investigation is needed using larger diverse samples.

